# Bioinformatics analysis of electroacupuncture treatment for ischemic stroke: exploring transcriptional regulatory mechanisms mediated by super-enhancers

**DOI:** 10.3389/fnins.2025.1522466

**Published:** 2025-03-05

**Authors:** Chunxiao Wu, Qizhang Wang, Zhirui Xu, Chuyu Deng, Chunzhi Tang

**Affiliations:** ^1^Shenzhen Hospital of Integrated Traditional Chinese and Western Medicine, Shenzhen, China; ^2^Shenzhen Clinical College of Integrated Chinese and Western Medicine, Guangzhou University of Chinese Medicine, Shenzhen, Guangdong, China; ^3^The Affiliated Traditional Chinese Medicine Hospital, Guangzhou Medical University, Guangzhou, Guangdong, China; ^4^Clinical Medical of Acupuncture, Moxibustion and Rehabilitation, Guangzhou University of Chinese Medicine, Guangzhou, Guangdong, China

**Keywords:** ischemic stroke, super-enhancers, electroacupuncture, core transcriptional regulatory circuitries, regulatory mechanisms, bioinformatics analysis

## Abstract

**Background:**

Ischemic stroke is a leading cause of disability and mortality, imposing substantial physical, emotional, and economic burdens on patients and society. This study aimed to explore the regulatory effects of super-enhancers (SEs) on gene expression in the context of ischemic stroke and their potential transcriptional regulatory mechanisms.

**Methods:**

Super-enhancers were identified via H3K27ac chromatin immunoprecipitation sequencing (ChIP-seq) and ROSE software. RNA-sequencing (RNA-seq) was employed to screen for differentially expressed genes. A comparative analysis of ChIP-seq and RNA-seq data initially identified SE target genes, followed by further screening of key core differentially expressed SE target genes via the random forest method. The identified core SE target genes were initially validated through immunofluorescence and immunoblotting techniques. Additionally, potential core transcriptional regulatory circuits were preliminarily screened via the Coltron algorithm.

**Results:**

We identified SE-associated genes in the ischemic stroke model and electroacupuncture-treated groups, revealing 41 genes uniquely regulated by SEs in the electroacupuncture group compared with 367 in the model group. Enrichment analyses revealed that pathways involved in axon guidance, regulation of lipolysis in adipocytes and sphingolipid signaling pathway were significantly enriched in the SE target genes, suggesting that these pathways may be involved in the therapeutic effects of electroacupuncture. Notably, HDAC7 emerged as a key SE-driven gene; its expression was significantly reduced following electroacupuncture treatment, indicating its potential as a therapeutic target. Protein expression analyses confirmed elevated levels of HDAC7 in the model group, which were reduced by electroacupuncture intervention (*p* < 0.05). Furthermore, core transcriptional regulatory circuitries involving SOX8, FOXK1, and KLF13 were identified, highlighting their roles in the modulation of SE-mediated gene regulation by acupuncture in the ischemic stroke context.

**Conclusion:**

Overall, our findings provide novel insights into the molecular mechanisms by which acupuncture may treat ischemic stroke, identifying key SE target genes and transcriptional circuits as promising targets for future therapeutic strategies. Further research is warranted to validate these findings in clinical settings and explore the translational potential of acupuncture in ischemic stroke treatment.

## Introduction

Ischemic stroke, is a leading cause of disability and mortality worldwide, making it a major global health concern. The pathophysiology of ischemic stroke involves the interruption of the blood supply to the brain, leading to neuronal cell death and subsequent brain infarction. There are multiple factors that impact the severity of this condition, including systemic blood pressure, the severity and duration of ischemia, and the location of infarcts, all of which complicate the clinical management of affected patients (Zhao et al., 2022). Current therapeutic strategies focus mainly on restoring blood flow; however, these interventions often fail to address the underlying biological mechanisms that promote neuronal injury and limit recovery (Aguiar de Sousa et al., 2019). This highlights the urgent need for innovative therapeutic approaches that not only target acute ischemic events but also improve long-term patient outcomes after ischemic injury.

Studies have increasingly focused on the molecular and cellular mechanisms that underlie ischemic stroke, including the roles of inflammatory responses and oxidative stress in excessive reactive oxygen species (ROS) production, which eventually results in neuronal damage (Jin et al., 2017; Chen et al., 2020). Moreover, emerging evidence suggests that specific signaling pathways, such as those involved in ferroptosis, mitosis, autophagy, necrosis, and apoptosis, are activated following ischemic events and may play critical roles in mediating angiogenesis and neuroprotection in the peri-infarct regions of the brain (Hatakeyama et al., 2020; Qin et al., 2022). Understanding these pathways is essential for developing targeted therapies that could mitigate the damaging effects of ischemia and promote recovery.

Despite advances in our understanding of ischemic stroke, there are significant gaps in knowledge regarding the genetic and epigenetic factors that influence stroke outcomes. Specifically, the regulation of gene expression through enhancers and super-enhancers has garnered attention, as these elements are pivotal in controlling the transcriptional programs that dictate cellular responses to ischemic stress (Thandapani, 2019; Martin et al., 2020). Super-enhancers, characterized by their enrichment of critical transcription factors, cofactors, and histones, are potent transcriptional regulatory elements with remarkable activity and specificity (Grosveld et al., 2021; Blayney et al., 2023). Furthermore, the literature indicates that super-enhancers are pivotal determinants of gene expression trends in neurological disorders, with histone modification changes induced by super-enhancers being intricately associated with conditions such as ischemic stroke, Parkinson’s disease, and Alzheimer’s disease (Carullo and Day, 2019). The role of super-enhancers in regulating genes associated with neuronal survival and repair mechanisms remains largely unexplored, presenting a valuable opportunity for future research to elucidate these connections and identify potential therapeutic targets.

In this context, acupuncture, particularly electroacupuncture, has been proposed as a complementary therapeutic modality for ischemic stroke rehabilitation. Preliminary studies indicate that acupuncture may influence gene expression and the regulatory networks involved in neuroprotection and recovery (Wu et al., 2018; Wu et al., 2021). As super-enhancers regulate gene expression, they may play a role in the regulation of genes related to ischemic stroke by electroacupuncture. However, the specific molecular mechanisms by which acupuncture affects gene regulation and super-enhancer activity in the context of ischemic stroke are poorly understood. These findings necessitate a systematic investigation into the relationships among acupuncture, super-enhancers, and gene expression in ischemic stroke models.

Here, high-throughput sequencing technologies, including RNA sequencing (RNA-seq) and chromatin immunoprecipitation sequencing (ChIP-seq), were used to comprehensively evaluate the impact of acupuncture on gene expression and the regulatory mechanisms involved in ischemic stroke. The identification of super-enhancer-associated genes and their core transcriptional circuits will provide insights into the molecular underpinnings of the therapeutic effects of acupuncture. Ultimately, this study aims to establish a theoretical foundation for acupuncture as a viable intervention in the management of ischemic stroke, potentially offering new avenues for personalized treatment strategies that align with the principles of precision medicine.

## Methods

### Ethics and consent

The Ethics Committee of Guangzhou University of Chinese Medicine approved all the experimental procedures in accordance with the Guide for the Care and Use of Laboratory Animals (Approval No. 20240301051).

### Laboratory animals and tissue collection

Adult male C57BL/6 mice (aged 8–10 weeks, weight between 20 and 25 g, SPF grade) were acquired from the Animal Experiment Center of Guangzhou University of Chinese Medicine. Initially, the mice were randomly allocated into two groups for ChIP-seq: the middle cerebral artery occlusion (MCAO) model group and the electroacupuncture group. The RNA-seq data were derived from our prior sequencing data (Wu et al., 2021), and the mice in this dataset were categorized into three groups: the sham model group, the MCAO model group, and the electroacupuncture group, with three mice in each category. The animals were subsequently randomly distributed into the following three groups (sham group, MCAO model group, and MCAO model + acupuncture group) to validate the core super-enhancer target genes identified from the integrated RNA-seq and ChIP-seq analysis and to preliminarily investigate the epigenetic modification mechanisms of super-enhancers regulated by electroacupuncture in ischemic stroke. All the mice were maintained under regulated temperature and humidity conditions with a 12-h light–dark cycle and unrestricted access to food and water.

### MCAO model

The MCAO model was established via the technique previously described (Engel et al., 2011; Bertrand et al., 2017). In brief, the mice were initially anesthetized via the inhalation of isoflurane to minimize discomfort. A midline incision was subsequently made under a stereomicroscope to expose the left common carotid artery, left internal carotid artery, external carotid artery, and vagus nerve. These structures were then separated and ligated as necessary. A suture plug measuring approximately 11 ± 0.5 mm in length was inserted into the internal carotid artery through the incision to prevent blood flow to the middle cerebral artery. One hour after ischemic occlusion, the suture plug was gradually withdrawn to restore the blood supply and induce ischemia–reperfusion injury. The mice in the sham surgery group underwent identical surgical procedures; however, MCAO was not performed. The criteria for evaluating the success of the MCAO model involve assessing the neurological function of animals using the Longa Score Scale (0–5 scales) (Longa et al., 1989). Mice in the MCAO model that receive a score ranging from 1 to 3 points are selected for inclusion in the study for further research. Comprehensive criteria for determining the success of the MCAO model, along with the Longa Score for each group, were detailed in Supplementary Table S1 and Supplementary Figure S1.

### Acupuncture method

The acupoints “Baihui” and “Dazhui,” along with “Zusanli” and “Quchi,” were selected as targeted intervention acupoints on the basis of animal acupoint selection criteria outlined in “Names and Locations of Acupoints Commonly Used in Laboratory Animals Part 3: Mice” (Acupuncture-Moxibustion, 2021). Following standard sterilization protocols using iodine and alcohol, a disposable sterile acupuncture needle (0.16 × 13 mm) was aseptically inserted into each designated acupoint by the operator. An electroacupuncture apparatus was subsequently connected: “Baihui” and “Dazhui” formed one group for electrical stimulation, whereas “Zusanli” and “Quchi” constituted a second group. The electrical wave applied was continuous with a current set at 1 milliampere and a stimulation frequency of 2 Hz (Chang et al., 2018; Xing et al., 2018); slight vibrations were used to assess the response of the tissue surrounding each acupoint during the treatment sessions. Each mouse received acupuncture intervention for 20 min per session over a period of 7 days.

### Behavioral assessment

An initial behavioral evaluation of the mice was conducted 1 day prior to the MCAO surgery, with a follow-up assessment performed 7 days post-surgery. The specific methodologies employed for these behavioral assessments are detailed below.

#### Adhesive removal test

The methodology for this behavioral assessment was adapted from established protocols (Hines and Haydon, 2013). In brief, a five-day training regimen was implemented prior to the formal evaluation to acclimate the mice to the laboratory environment. Adhesive tape was then affixed to the affected forelimb of each mouse. During the assessment, the time taken by the mice to remove the adhesive tape from their wrist were recorded. Mice were given a maximum of 300 s to complete the adhesive tape removal. Behavioral evaluations for all experimental groups were conducted both prior to the MCAO procedure and 7 days following the administration of EA treatment.

#### Grid walking test

The grid walking test, commonly referred to as the foot fault test, was executed in accordance with previously documented methodologies (Schaar et al., 2010). In brief, mice were positioned on an elevated grid with 15 mm-by-15 mm openings, allowing them to move the grid freely for a period of 300 s. A foot fault was recorded each time a paw slipped or fell through a grid bar. The total number of steps taken by the mice while navigating the grid was documented. The foot fault index for the affected forelimb in each group was calculated using the formula: (number of foot faults/total number of steps) × 100%. Prior to the test, mice were acclimatized to the grid, and assessments were conducted both before the MCAO surgery and 7 days post-treatment.

### Histological and morphological assessment

All mice underwent careful anesthesia, followed by cardiac perfusion with chilled phosphate-buffered saline (PBS), and were subsequently fixed in a 4% formaldehyde solution. The brains were promptly extracted and subjected to overnight fixation in the same 4% formaldehyde solution at 4°C. Dehydration was then performed using a sucrose gradient. From each brain, with a primary focus on the striatum, for immunofluorescence analysis to evaluate the infarcted area. Specifically, the sections were incubated overnight at 4°C with a rabbit anti-NeuN polyclonal antibody (Proteintech, United States; 1:500), followed by incubation at room temperature with a goat anti-rabbit secondary antibody (Abcam, United States; 1:1000). Imaging was conducted using a Nikon confocal microscope. The percentage of the infarcted area within the mouse brain was quantified using the following formula: Infarct area % = (Area of neuronal loss in each section)/(Total brain area of each section) × 100%. Additionally, to identify and quantify the surviving neuronal cells, fluorescent images of the tissue sections were captured and analyzed using a confocal microscope. Image J software was employed to determine the number of positive neuronal cells in both the affected cortical and striatal regions.

### RNA-seq and analysis of differentially expressed genes

In brief, RNA was extracted from brain tissue via TRIzol, and quality was assessed with a NanoDrop and Agilent Bioanalyzer. mRNA was purified with oligo(dT) beads and converted to cDNA, followed by library construction per the manufacturer’s standards. The PCR products were denatured and circularized to create the final library, which was amplified to produce DNA nanoballs (DNBs). DNBs were loaded onto a nanoarray for single-end 50-base reads on the BGISeq500 platform. Transcript sequences were normalized to fragments per kilobase of transcript per million mapped reads (FPKM), and differential gene expression was analyzed with DESeq2. Genes with Q ≤ 0.05 or FDR ≤ 0.001 were identified as differentially expressed genes (Cheng et al., 2019). The fold change (FC) value was employed to distinguish between upregulated and downregulated genes.

### ChIP-seq

Brain tissue samples (2–3 g) from each group were collected and placed in a PBS solution. The samples were subsequently ground and filtered through a cell strainer. The samples were treated with 1% formaldehyde to induce cross-linking, followed by the addition of glycine to terminate the cross-linking reaction. The cross-linked DNA–protein complexes were sonicated, after which agarose gel electrophoresis was performed for detection. First, protein G beads were washed before the addition of ChIP dilution buffer and the target protein antibody H3K27ac to form the antibody-target protein–DNA complex. Multiple washes were conducted to remove non-specifically bound chromatin and purify the complex. Crosslinking was then reversed, and the resulting DNA fragments were purified. Finally, the blunt ends of the DNA fragments were repaired, followed by the ligation of sequencing adapters. The PCR products were amplified, and selected fragments were used to construct the sequencing library, which was subsequently sequenced via the Illumina NovaSeq 6,000 PE150 platform.

### Identifying ChIP-seq-enriched regions

Cutadapt (v2.5) was used to trim adapters and filter out low-quality sequences. The sequencing reads were aligned to the mouse genome mm10 with Bowtie2 (v2.3.5.1) via default parameters. Regions of H3K27ac enrichment were calculated with MACS2 (v2.1.2) using the parameters m 5 50. The MACS peaks of H3K27ac served as enhancers for identifying super-enhancers.

### Definition of enhancers and super-enhancers

The enhancers were concatenated, and super-enhancers were discerned via ROSE^1^ as previously described (Whyte et al., 2013). In summary, this algorithm merges constituent enhancers if they lie within a specified distance and ranks the enhancers on the basis of their input-subtracted H3K27ac signal. It subsequently distinguishes super-enhancers from typical enhancers by pinpointing an inflection point of the H3K27ac signal in relation to the enhancer rank. ROSE was executed with a stitching distance of 12,500 bp, meaning that we permitted enhancers within 12,500 bp to be amalgamated. Furthermore, peaks within ±2,500 bp of an annotated transcription start site were omitted from stitching. For enhancer annotation, we utilized the homer (v4.10.4) utility annotatePeaks.pl, which links peaks to their adjacent genes.

### Gene ontology (GO) and Kyoto Encyclopedia of Genes and Genomes (KEGG) pathway enrichment analyses

The GO and KEGG pathway enrichment analyses of the genes linked to super-enhancers were conducted via the clusterProfiler R package (v3.6.0). A *p*-value ≤0.05 was considered indicative of enriched GO terms and KEGG pathways.

### Identification of key super-enhancer regulatory genes in ischemic stroke regulated by acupuncture

Key super-enhancer-driven genes were identified via the random forest algorithm, with core genes selected via the R package “randomForest.” To find the optimal error rate and the ideal number of stable trees, we calculated the error rate for each tree from 1 to 500. The random forest method was subsequently employed to screen for key super-enhancer-driven genes, utilizing the minimum Gini (MDG) in the random forest algorithm to assess classification accuracy and dimensional significance values. The top genes that presented the highest MDG scores were designated core super-enhancer genes. Furthermore, the Friends analysis method is extensively utilized for the identification of critical genes. In order to pinpoint critical core super-enhancer genes, the R package GOSemSim (Yu, 2020) was employed to compute the functional correlations among the primary super-enhancer-driven genes. The genes with the highest rankings were designated as key super-enhancer genes. Subsequently, we conducted an intersection analysis of the top three significant genes identified by both the random forest and Friends analysis methods to determine the core super-enhancer-driven genes. Additionally, the signal peaks of H3K27ac associated with key super-enhancer-target genes were visualized and analyzed utilizing the Integrative Genomics Viewer (IGV, version 2.16.2).

### Screening of the super-enhancer–transcription factor regulatory network in ischemic stroke

The Coltron algorithm we devised was employed to identify essential regulatory circuits, utilizing H3K27ac ChIP-seq read data aligned with the mouse genome, along with the ChIP-seq peaks generated by MACS (Zhang et al., 2008) and an enhancer list generated by ROSE (see Footnote 1) (Whyte et al., 2013). The process of identifying core transcriptional regulatory circuits (CRC) is as follows: (1) determine the nearest active genes using the outcomes from the super-enhancer regions and the alignment results; (2) exclude the segment of the nearest active genes that function as transcription factors and identify the transcription factors governed by super-enhancers; (3) gather transcription factor motifs from the TRANSFAC and JASPAR databases; and (4) utilize FIMO in MEME62 to examine the sequences of super-enhancer elements for motifs. The *p*-value threshold for FIMO is established at 1*10^^−4^. (5) When a transcription factor is capable of binding to the super-enhancer element that regulates it, it is classified as a self-regulating transcription factor. Transcription factors assigned to super-enhancers, whose component set encompasses at least three instances of DNA sequence motifs corresponding to their own protein products, are designated self-regulating transcription factors. From the group of self-regulating transcription factors, those capable of binding to the super-enhancers of other self-regulating transcription factors are predicted via the same criteria outlined above. All the possible fully interconnected self-regulating circuits are subsequently constructed through recursive identification. After multiple configurations of fully interconnected self-regulating circuits of transcription factors are established, the most representative fully interconnected self-regulating circuit is chosen as the CRC model (Saint-Andre et al., 2016; Feng et al., 2023).

### Immunoblotting and immunofluorescence

Damaged tissues from each group of mice were frozen in liquid nitrogen and then pulverized to prepare protein extracts. A specific amount of primary antibody targeting the gene of interest was added to the lysed tissues for western blotting (WB) protein quantification. Brain sections from the affected areas of each group of mice were subjected to immunofluorescence. A primary antibody from rabbits that targets the gene of interest was added to the brain tissue sections. This was followed by the addition of a corresponding fluorescent secondary antibody for immunofluorescence. The fluorescence of the target gene was examined via laser confocal microscopy, and the expression levels in each group were quantified with Fiji ImageJ^2^.

### Statistical analysis

The data were analyzed via GraphPad Prism 9 (GraphPad Software, San Diego, CA, United States) and R version 4.2.1^3^. For normally distributed data with homogeneous variances across groups, the results are presented as the means ± standard errors of the means, and one-way ANOVA was conducted, accompanied by Bonferroni correction for *post hoc* comparisons. In instances where variances were not homogeneous, Dunnett’s T3 test was employed; a *p*-value of less than 0.05 was considered statistically significant.

## Results

### EA enhances motor function following cerebral ischemic injury

**Figure 1:**
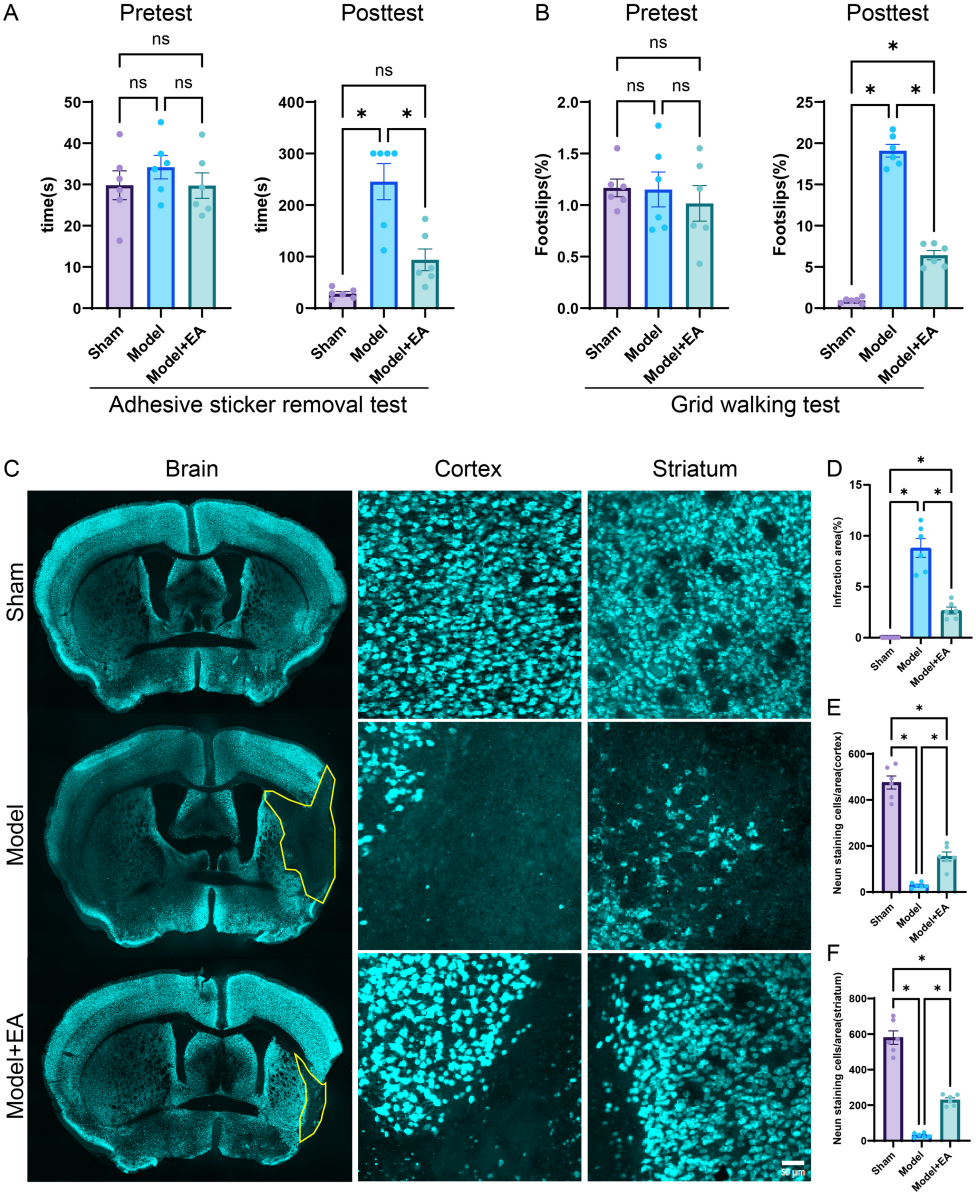
Effects of EA on neurological impairments in MCAO mice. **(A)** The time required to remove adhesive stickers was measured for the three groups both before MCAO surgery and after a 7-day EA intervention (*n* = 6 mice per group). **(B)** The proportion of foot-faults in the affected forelimb was assessed in each group prior to MCAO surgery and following a 7-day EA treatment protocol (*n* = 6 mice per group). **(C)** Representative sections stained with NeuN from each group after 7 days post-ischemic stroke is depicted. **(D)** The percentage of cerebral infarction area in the brains of mice across the groups is presented (*n* = 6 slices per group). **(E,F)** Quantitative analysis of NeuN-positive cells in the cortex and striatum was conducted (*n* = 6 slices per group). Data are presented as means ± SEM. *p < 0.05, ^ns^*p* > 0.05.

Before the commencement of MCAO surgery, there were no significant differences in the time required to remove the adhesive tape among the three experimental groups, suggesting that the groups were comparable at baseline (all *p* > 0.05). After a period of 7 days, the electroacupuncture group exhibited a significantly shorter duration for tape removal compared to the MCAO model group, indicating improved motor function in the electroacupuncture-treated mice. This observation suggests that electroacupuncture may mitigate neurological impairments and enhance motor performance following ischemic stroke (Figure 1A). The foot slip test is utilized as a primary measure of limb motor coordination in the context of ischemic stroke. Prior to MCAO surgery, statistical analyses indicated no significant differences in foot slip percentages among the three groups of mice, confirming their comparable baseline characteristics (*p* > 0.05). Seven days post-injury, the MCAO + EA group showed a reduced incidence of foot slips compared to the MCAO control group, with statistically significant differences observed (*p* < 0.05, Figure 1B). This suggests that the intervention may have a beneficial effect in improving motor coordination in mice subjected to MCAO.

### Electroacupuncture mitigates pathological damage after cerebral ischemic injury

As demonstrated in Figure 1C, the MCAO model exhibited significant neuronal loss compared to the sham surgery group. However, after a seven-day treatment period, electroacupuncture significantly decreased the extent of infraction areas relative to the MCAO model group (*p* < 0.05). Furthermore, neuronal populations within the brain tissue were evaluated using NEUN staining. The ischemic brain injury model was associated with neuronal loss in the cortical and striatal regions. Nonetheless, electroacupuncture treatment resulted in a significant reduction in neuronal loss in both the cortical and striatal regions compared to the model group (*p* < 0.05) (Figures 1C–F). Therefore, electroacupuncture treatment appears to promote neuronal survival following cerebral ischemic injury, providing neuroprotective effects.

### Identification of super-enhancer-associated genes in ischemic stroke

**Figure 2:**
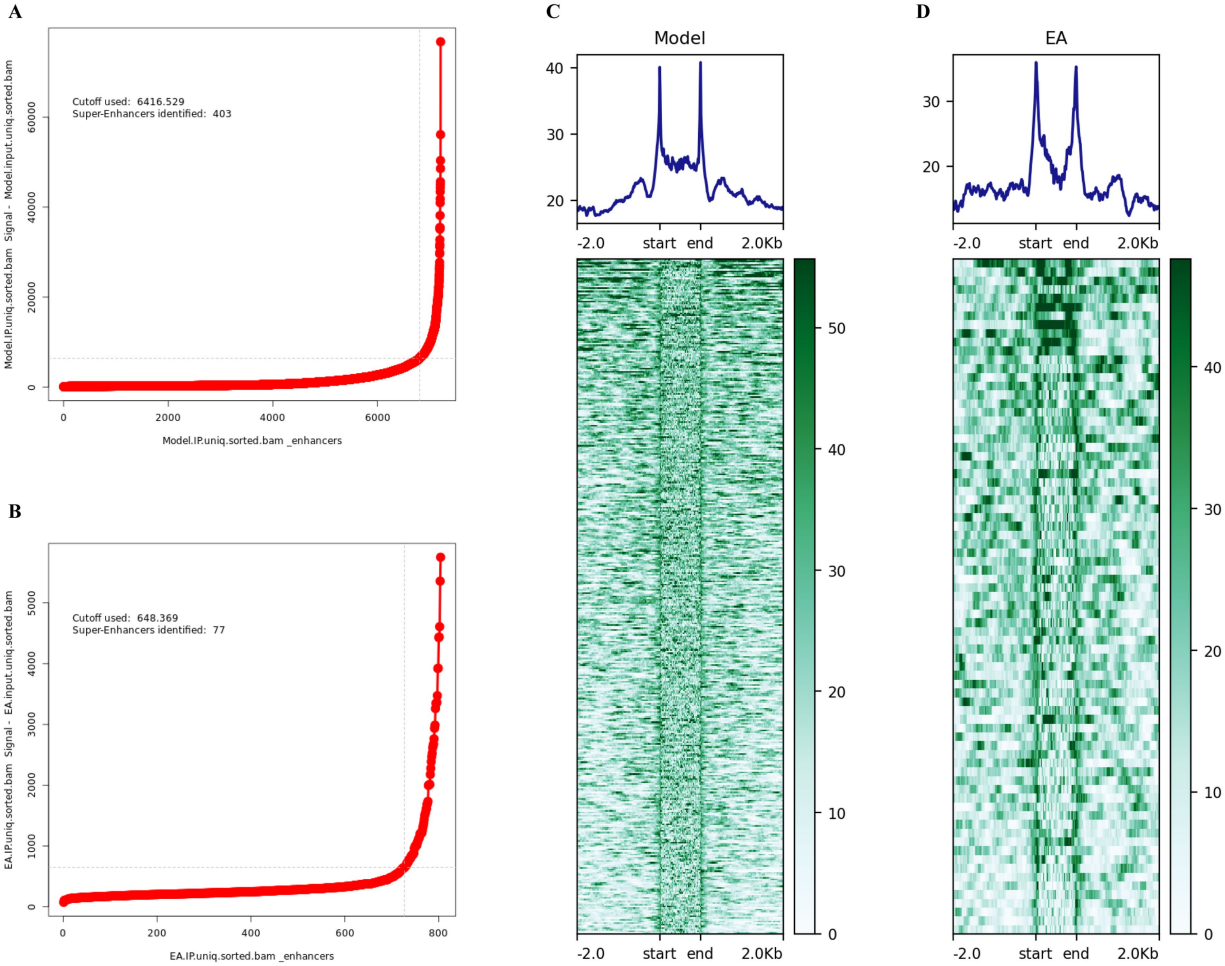
Identification of super-enhancers in ischemic stroke. **(A)** Visualization of super-enhancers identified in the MCAO model group, based on ranked H3K27ac signal intensity using the ROSE algorithm. A total of 403 super-enhancers were identified. **(B)** Visualization of super-enhancers identified in the EA+MCAO model group, based on ranked H3K27ac signal intensity using the ROSE algorithm. A total of 77 super-enhancers were identified. **(C)** Heatmap and aggregate plots depicting H3K27ac ChIP-seq signals within ±2 kb of peak centers in the MCAO model group. **(D)** Heatmap and aggregate plots of H3k27ac ChIP-seq signals at ±2 kb of peak centers in the EA + MCAO model group.

This study aimed to investigate potentially differentially regulated super-enhancer-driven genes modulated by electroacupuncture in ischemic stroke through ChIP-seq analysis. Considering that super-enhancers play crucial cis-regulatory roles within cells, we utilized the ROSE tool to identify super-enhancers and predict their nearest associated genes on the basis of chromosomal spatial positioning. In total, 77 super-enhancer-associated genes were identified in the electroacupuncture intervention group, whereas 403 genes were detected in the MCAO model group (Figures 2A,B). DeepTools software was employed to analyze and visualize the signal intensity across super-enhancer regions for each experimental group (Figures 2C,D).

### KEGG/GO analysis of target genes regulated by super enhancers

**Figure 3:**
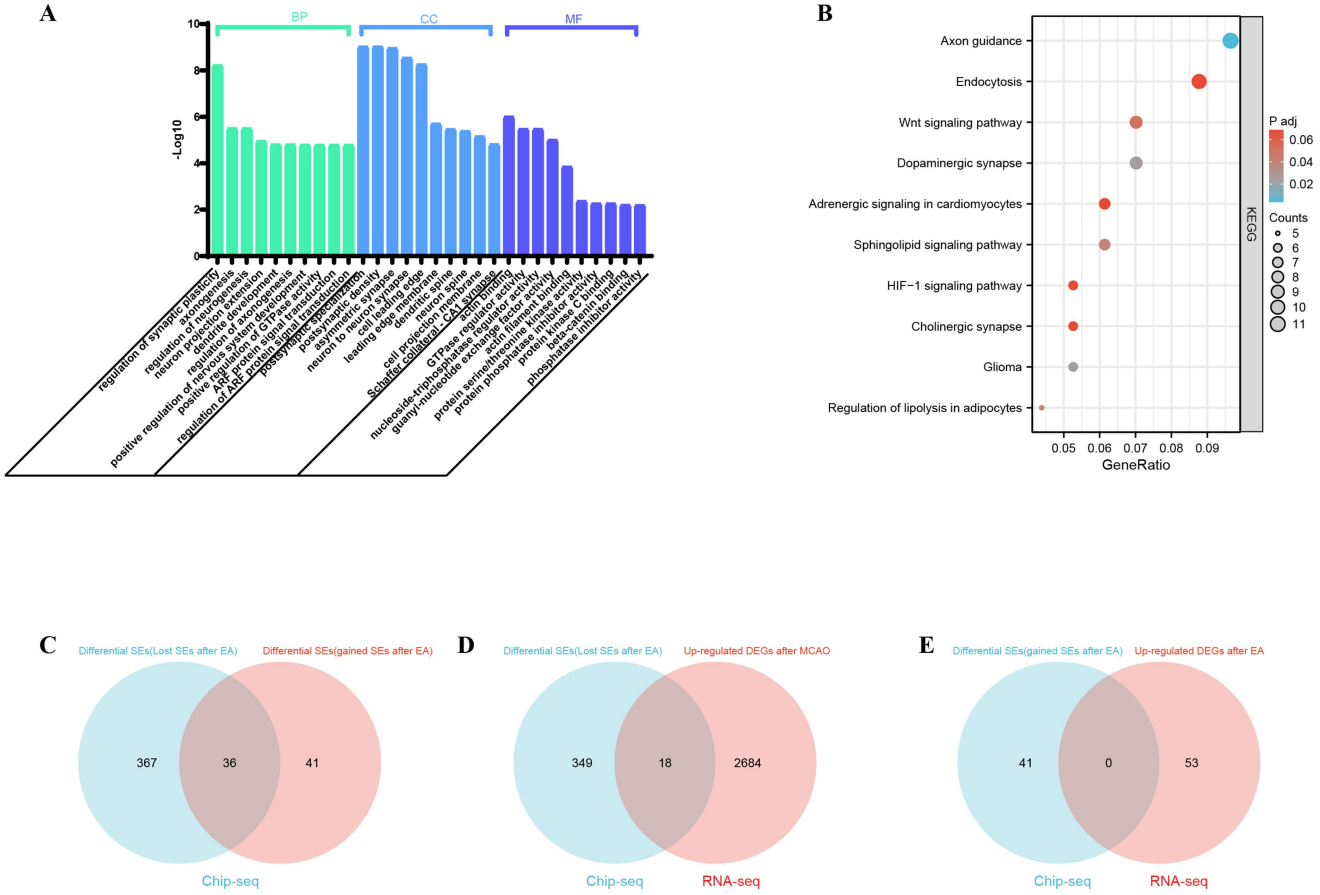
Functional enrichment analysis of super-enhancer related genes and analysis of potential super-Enhancer targeted Genes in ischemic stroke. **(A)** The biological processes (BP), cellular components (CC), and molecular functions (MF) associated with SE-related genes were compared between the MCAO model group and the EA+MCAO model group. **(B)** Kyoto Encyclopedia of Genes and Genomes (KEGG) pathways of SE-related genes were examined to identify differences between the MCAO model group and the EA+MCAO model group; *p* < 0.05 was set as the cutoff criteria. Analysis of Potential Super-Enhancer (SE) Targeted Genes in Ischemic Stroke: **(C)** the Venn diagram illustrates the differential SEs between the Model group and the EA + MCAO model group. **(D)** The Venn diagram illustrates the intersection between differential SE-related genes (lost SEs after EA) and upregulated differentially expressed genes (DEGs) within the model group in RNA-Seq analysis. **(E)** The Venn diagram depicts the intersection between differential SE-related genes (gained SEs after EA) and upregulated DEGs subsequent to EA intervention in RNA-Seq analysis.

The GO and KEGG pathway analyses were performed to examine the differentially super-enhancer target genes between the experimental group (EA) and the model group. The results revealed significantly enriched BP terms, including ‘regulation of synaptic plasticity,’ ‘axon genesis,’ ‘regulation of neurogenesis,’ ‘neuron projection extension,’ and ‘dendrite development’. Additionally, GO cellular component (CC) enrichment analysis revealed significantly enriched GO CC terms such as ‘postsynaptic specialization,’ ‘postsynaptic density,’ ‘asymmetric synapse,’ ‘neuron to neuron synapse,’ and ‘cell leading edge’. The top five enriched GO molecular function (MF) terms included ‘actin binding,’ ‘GTPase regulator activity,’ ‘nucleoside-triphosphatase regulator activity,’ ‘guanyl-nucleotide exchange factor activity,’ and ‘actin filament binding’. Furthermore, KEGG signaling pathways such as Axon guidance, Dopaminergic synapse, Glioma, Regulation of lipolysis in adipocytes, Sphingolipid signaling pathway, Wnt signaling pathway, Endocytosis and HIF-1 signaling pathway were significantly enriched in super-enhancer-related genes. The specific details are shown in Figures 3A,B.

### Integrated ChIP-seq and RNA-seq analysis

Since super-enhancers are associated with gene transcription, genes that exhibit abnormal expression may represent key pathogenic factors in various diseases. The comparison of super-enhancer-driven genes between the model group and the electroacupuncture group revealed that 41 genes were uniquely regulated by super-enhancers in the electroacupuncture group [namely differential SE-related genes (gained super-enhancers after EA)], whereas 367 genes were regulated solely by super-enhancers in the model group [namely differential SE-related genes (lost super-enhancers after EA)] (Figure 3C). The genes regulated by super-enhancers in the electroacupuncture group, along with those lost super-enhancers by electroacupuncture compared with those in the model group, may serve as potential target genes for electroacupuncture. Super-enhancers facilitate gene expression by enriching transcription factors, which leads to the upregulation of critical genes. We compared the upregulated genes with those associated with super-enhancers. RNA sequencing analysis revealed the expression profiles of the differentially expressed genes within each group, revealing 2,702 upregulated genes in the model group and 53 upregulated genes in the electroacupuncture group. The analysis identified key regulatory genes associated with super-enhancers by examining the overlap between differentially expressed super-enhancer-related genes and upregulated genes in RNA-seq data. Specifically, this involved two comparisons: first, the intersection of genes associated with lost super-enhancers following EA with those upregulated in the model group relative to the EA group; and second, the intersection of genes associated with gained super-enhancers post-EA with those upregulated following the electroacupuncture intervention. Notably, the analysis revealed no overlapping genes between the super-enhancers gained post-EA and those upregulated in the RNA-seq data following the EA intervention. However, a comparison of genes associated with lost super-enhancers post-EA and upregulated genes in the MCAO group from RNA-seq identified 18 overlapping differentially expressed genes, which may serve as super-enhancer target genes, potentially exerting a suppressive role in the regulation of ischemic stroke via electroacupuncture. And these 18 selected super-enhancer-driven differentially expressed genes underwent were further analyzed and validated to confirm their significance (Figures 3D,E and Supplementary Table S2).

### Potential super-enhancer target genes associated with ischemic stroke regulated by acupuncture as analyzed by random forest machine learning

**Figure 4:**
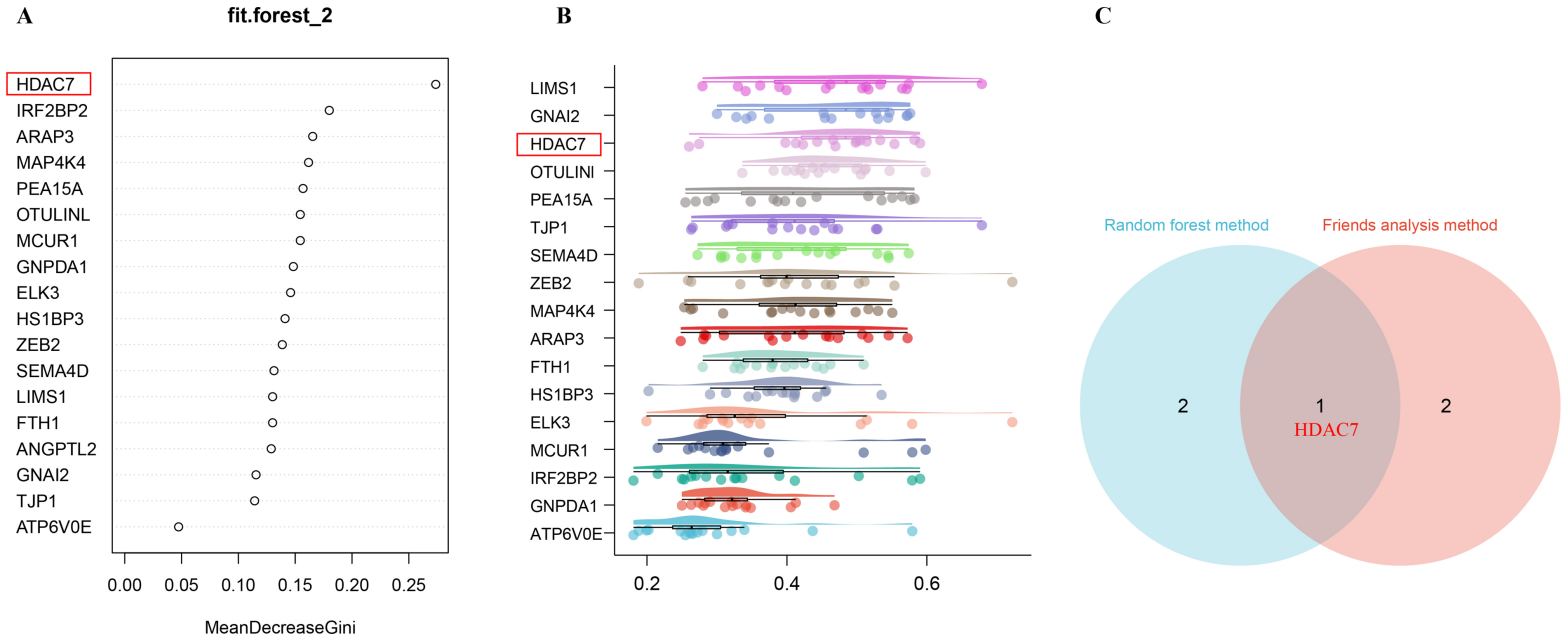
Identification of key regulatory genes associated with super-enhancers in ischemic stroke, modulated by acupuncture, through the application of machine learning algorithms. **(A)** The feature importance plot, evaluated using the Gini index within a Random Forest machine learning model, demonstrates that higher MeanDecreaseGini values indicate a greater ranking and increased significance. **(B)** The Friends analysis method constructs a gene interaction network and evaluates the significance of each gene through the examination of network topology. The box plot illustrates that genes are ranked in descending order according to their mean similarity to other genes. The genes with the highest rankings exhibit the greatest similarity to other genes, thereby identifying them as key regulatory genes associated with super-enhancers. **(C)** The Venn diagram illustrates the core super-enhancer regulatory genes identified from the intersection of the top three significant super-enhancer-driven genes, as ranked by random forests and Friends analysis. The gene HDAC7 emerges as the core super-enhancer regulatory gene from the intersection of these two analytical approaches.

The significance ranking of 18 differentially expressed super-enhancer-driven genes was determined via random forests, with the top-ranked super-enhancer-driven genes identified as “HDAC7,” followed by “ARAP3” and “IRF2BP2,” as illustrated in Figure 4A. The Friends analysis revealed that HDAC7 exhibited a robust correlation with other super enhancer-driven genes and may therefore play a pivotal role in ischemic stroke (Figure 4B). Given that super-enhancers are intricately linked to histone acetylation, and the intersection of random forests and Friends analysis indicated that HDAC7 is a crucial super-enhancer-driven gene (Figure 4C). The histone deacetylase HDAC7, upregulated by super-enhancers, is highly expressed in MCAO model mice according to the RNA-seq data, leading to the preliminary hypothesis that HDAC7 could be a pivotal pathogenic factor in ischemic stroke. Moreover, electroacupuncture may address ischemic stroke by modulating the expression of HDAC7, which is influenced by super-enhancers (Figure 4).

### Validation of potential super-enhancer-associated genes

**Figure 5:**
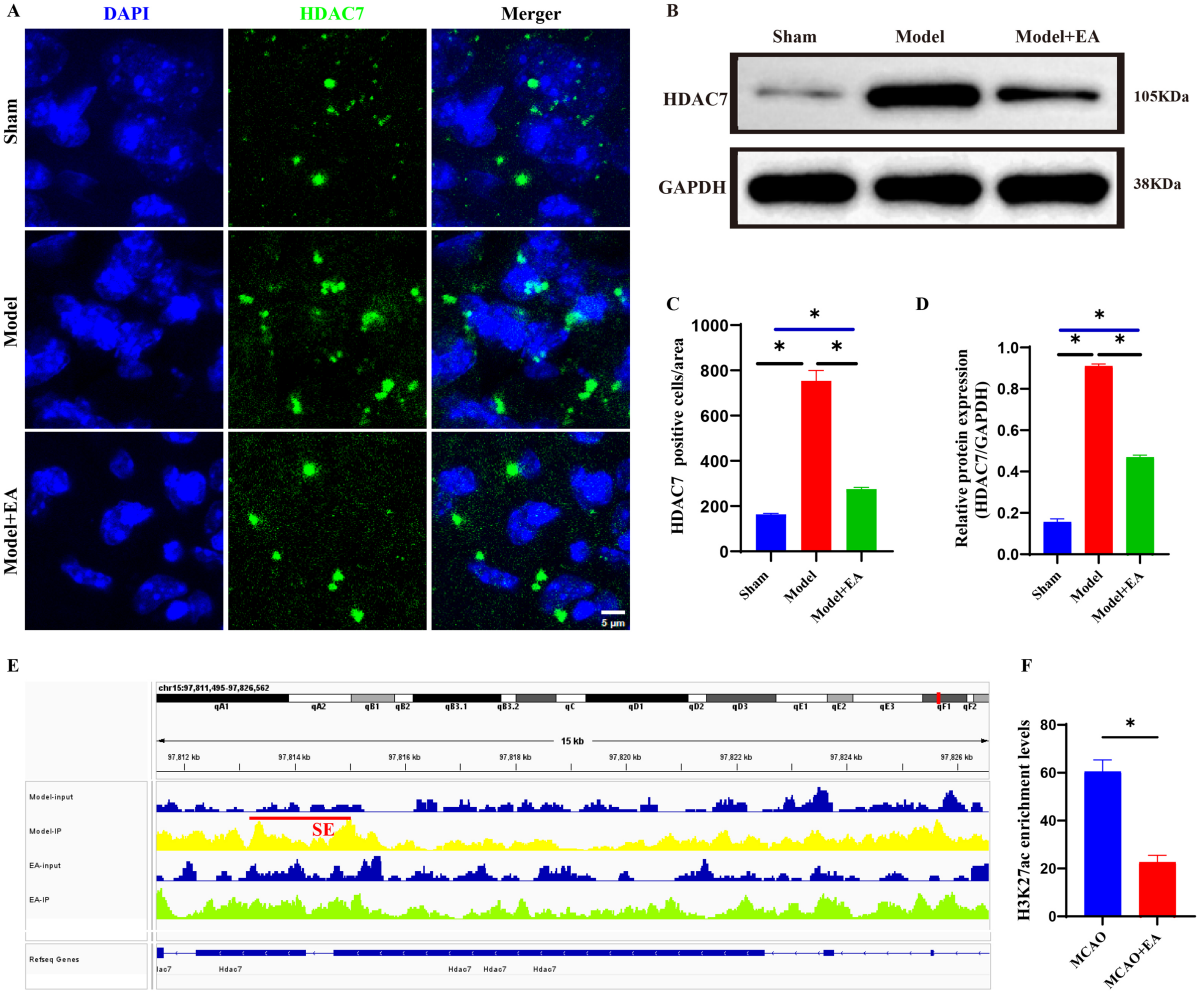
Validation of potential super-enhancer-associated gene (HDAC7) via WB and immunofluorescence. **(A)** Representative images of immunofluorescence staining of HDAC7 expressions (Green) in the ischemic stroke. Scale bar = 10 μm. **(B)** Protein expression of HDAC7 gene. **(C)** Statistic analysis of the number of HDAC7+ cells per field in the injury region of ischemic mice. Sham: the Sham MCAO model group, Model: the MCAO model group, EA + Model: the electroacupuncture + MCAO model group. Data were expressed as mean ± standard error; *n* = 6 slices from 3 mice per group. *p < 0.05, ^ns^*P* > 0.05. **(D)** Quantitative analyses of HDAC7 protein expression according to groups. Sham: the Sham MCAO model group, Model: the MCAO model group, EA + Model: the electroacupuncture + MCAO model group. Data were expressed as mean ± standard error; *n* = 3 per group. *p < 0.05, ^ns^*P* > 0.05. **(E)** Integrative genomics viewer (IGV) plots showing enrichment of H3K27ac peaks of HDAC7 gene using aggregate ChIP-Seq profiles of ischemic brain tissue from the model and EA groups. Red line indicated the SE loci. **(F)** Quantitative analysis of the enrichment levels of H3K27ac peaks associated with the HDAC7 gene between the Model and EA groups. Data are presented as mean ± standard error; *n* = 6 peak values. *p < 0.05, ^ns^*P* > 0.05.

Through WB and immunofluorescence, the expression of HDAC7 regulated by super enhancers in both the model group and the electroacupuncture group was confirmed. The findings revealed that the protein and fluorescence expression levels of HDAC7 were significantly greater in the model group than in the electroacupuncture group and the sham surgery group (*p* < 0.05). Initial validation suggested that HDAC7 was aberrantly overexpressed in the ischemic stroke group, whereas electroacupuncture intervention had a suppressive effect on the fluorescence intensity and protein level of HDAC7 (Figures 5A–D). Furthermore, the IGV plot and H3K27ac peak value analysis revealed that H3K27ac (a marker for super-enhancers) levels were significantly more enriched in the model group than in the electroacupuncture group (Figures 5E,F).

### Screening of the super-enhancer–transcription factor regulatory network in ischemic stroke

**Figure 6:**
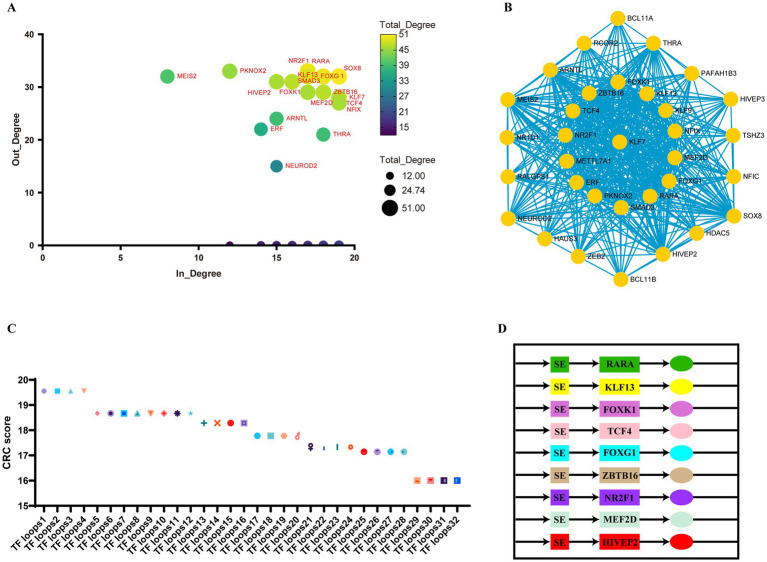
Core transcriptional regulatory circuits in ischemic stroke. **(A)** A scatter plot illustrating the in-degree (the number of transcription factors binding to the super-enhancer of a node gene) and out-degree (the number of super-enhancers bound by a node transcription factor) of the core regulatory circuits in ischemic stroke. **(B)** A network diagram depicting the interactions between transcription factors and super-enhancers in ischemic stroke. The size of each node is proportional to its total degree, and the edges shown in turquoise represent predicted interactions between transcription factors and super-enhancers. **(C)** A scatter plot displaying the scores of each core regulatory circuit, where a higher score indicates a superior ranking. Detailed information on transcription factor loops 1 through 32 is provided in Supplementary Table S2. **(D)** A CRC model established by top-ranked core transcription factors.

SEs and open chromatin regions are frequently enriched with specific transcription factors (TFs) that collaboratively regulate cellular transcription. To this end, we developed a super-enhancer-mediated transcriptional regulatory model for ischemic stroke by integrating H3K27ac data (Figure 6A). Using ChIP-seq experimental data to predict core transcription factors, we calculated the total degree (including both in-degree and out-degree) of genes encoding CRCs through machine learning. Genes with a relatively high total degree are considered potential core transcription factors. The primary core transcription factors included SOX8, KLF13, FOXK1, FOXG1, RARA, NR2F1, SMAD3, ZBTB16, and KLF7 (Figure 6B). The following sets were among the top-ranked CRCs identified by machine learning: [‘RARA,’ ‘KLF13,’ ‘TCF4,’ ‘FOXG1,’ ‘ZBTB16,’ ‘NR2F1,’ ‘FOXK1,’ ‘MEF2D,’ ‘HIVEP2’], [‘RARA,’ ‘KLF13,’ ‘SMAD3,’ ‘FOXG1,’ ‘ZBTB16,’ ‘NR2F1,’ ‘FOXK1,’ ‘MEF2D,’ ‘HIVEP2’], [‘SOX8,’ ‘KLF13,’ ‘TCF4,’ ‘FOXG1,’ ‘ZBTB16,’ ‘NR2F1,’ ‘FOXK1,’ ‘MEF2D,’ ‘HIVEP2’], [‘SOX8,’ ‘KLF13,’ ‘SMAD3,’ ‘FOXG1,’ ‘ZBTB16,’ ‘NR2F1,’ ‘FOXK1,’ ‘MEF2D,’ ‘HIVEP2’] (Figure 6C). These factors may represent key regulatory circuits of core transcription factors influencing pathogenic genes in the MCAO model (Figure 6D). Detailed information is presented in Figure 6 and Supplementary Table S3.

## Discussion

Through a series of behavioral assessments and pathological examinations, we have corroborated that EA facilitates the recovery of neurological function subsequent to an ischemic stroke. Additionally, EA has been observed to mitigate neuronal loss associated with ischemic stroke. Nonetheless, the intricate molecular mechanisms that underpin the effects of EA in the context of ischemic stroke remain inadequately elucidated. In this study, we focused on the pathophysiological mechanisms underlying ischemic stroke and the potential therapeutic effects of electroacupuncture. Using high-throughput sequencing and bioinformatics approaches, we aimed to elucidate the regulatory effects of electroacupuncture on the expression of genes associated with super-enhancers in ischemic stroke models. Our key findings indicate that electroacupuncture may modulate the expression of critical genes linked to super-enhancers, notably HDAC7, which could serve as potential therapeutic targets. These insights provide a novel perspective on the molecular mechanisms by which electroacupuncture treats ischemic stroke and contribute to the growing body of evidence supporting its therapeutic efficacy.

The interaction between super-enhancers and gene expression is pivotal for understanding the regulatory mechanisms underlying ischemic stroke and the therapeutic effects of electroacupuncture. Our findings revealed that the absence of super-enhancer-driven upregulated genes in the electroacupuncture group indicates significant modulation of gene regulatory networks in response to treatment. Compared with those in the electroacupuncture group, the overexpression of these super-enhancer-driven genes in the model group may be key regulatory targets of electroacupuncture, potentially exerting protective effects against ischemic stroke by inhibiting the expression of these genes. This observation suggests that electroacupuncture may alter the activity of super-enhancers, which are known to regulate gene expression dynamically, thus impacting gene transcription in a manner that mitigates the pathological effects of ischemic stroke (Blayney et al., 2023). The role of super-enhancers in facilitating gene expression is well documented, as they cluster transcriptional enhancers that drive the expression of genes critical for cell identity and function (Hnisz et al., 2013; Ryu et al., 2019). Furthermore, the interplay between super-enhancers and transcription factors can lead to complex regulatory landscapes that are essential for maintaining cellular homeostasis during ischemic conditions (Wilflingseder et al., 2020). Previous studies have shown that super-enhancers play an important role in inflammation, immunity, and neuronal degeneration (Pott and Lieb, 2015). The 3D chromatin structure of super-enhancers changes quickly in response to inflammatory stimuli, resulting in abnormal expression of inflammatory genes (Fanucchi et al., 2013; Higashijima et al., 2020). Ischemic stroke is closely linked to intracerebral inflammation, and super-enhancers, along with their related genes, are highly susceptible to enhancer factor suppression. Thus, inhibiting super-enhancer activity may offer therapeutic benefits in the response to intracerebral inflammation during ischemic stroke (Higashijima and Kanki, 2022). Therefore, elucidating the mechanisms by which electroacupuncture influences super-enhancer activity may provide insights into novel therapeutic strategies for the management of ischemic stroke.

The gene expression analysis conducted in this study provides compelling evidence for the role of specific genes, such as HDAC7, in the pathophysiology of ischemic stroke and the therapeutic effects of electroacupuncture. The significant downregulation of HDAC7 in the electroacupuncture group compared with the model group underscores the potential of electroacupuncture to modulate epigenetic regulation through super-enhancer dynamics. HDAC7, a class II histone deacetylase, has been implicated in various cellular processes including cell differentiation and survival, making it a critical target in stroke intervention (Demyanenko and Sharifulina, 2021). Research has shown that abnormally high expression of HDACs can lead to dysfunction of brain microvascular endothelial cells, enhanced oxidative stress and inflammatory responses, apoptosis, and cell death in an ischemic stroke model (Xu et al., 2020). Previous studies have also indicated that inhibitors of class II HDACs can effectively protect the brain from ischemic injury. They can induce angiogenesis, neurogenesis, and stem cell migration to damaged brain areas to promote functional recovery after cerebral ischemia, which is in line with the effects of electroacupuncture in our study (Demyanenko et al., 2021). Another study also revealed that electroacupuncture can mediate the transcription of related genes in an MCAO model by regulating the acetylation levels of H3K9/H3K27 at the promoters of apoptosis-related genes, thereby exerting neuroprotective effects against ischemia–reperfusion (I/R) injury (Meng et al., 2024). These findings are consistent with our findings, which also suggest that electroacupuncture regulates histone acetylation/deacetylation levels in ischemic stroke. The observed changes in gene expression not only reflect alterations in the histone acetylation landscape but also suggest a broader impact on the transcriptional networks governing neuronal survival and recovery poststroke. Furthermore, the absence of super-enhancer-driven upregulated genes in the electroacupuncture cohort highlights the need for further investigation into the specific pathways and interactions that are modulated by this treatment method, particularly regarding their implications for neuronal protection and functional recovery in ischemic stroke (Kim et al., 2020).

The identification of super-enhancer-driven genes, specifically “ARAP3,” and “IRF2BP2,” as potential targets in ischemic stroke aligns with previous studies that highlighted the role of these genes in neuroinflammatory processes and cellular responses to ischemic injury. The roles of ARAP3 and IRF2BP2 in cellular signaling and transcriptional regulation further support their potential relevance in stroke pathology, as they are known to participate in inflammatory cell activation and macrophage polarization, which are crucial for maintaining cellular homeostasis during ischemic conditions and play critical roles in neuronal survival during ischemic events (Cruz et al., 2017; McCormick et al., 2019; Lee et al., 2023; Zhang et al., 2023).

Pathway analysis has identified a notable enrichment of biological processes influenced by electroacupuncture, particularly those associated with synaptic plasticity, with axon guidance playing a crucial role in neuronal development and repair. Research suggests that enhancing axon guidance may improve neuronal survival and regeneration following ischemic stroke, indicating that therapies targeting this pathway could promote neurogenesis and synaptic remodeling, thereby facilitating recovery after cerebral ischemia (Luo et al., 2022; Mu et al., 2023; Weber et al., 2024). Furthermore, studies have shown that the expression of HDAC is associated with this signaling pathway, and modulating HDAC levels may support nerve cell regeneration after ischemic stroke and enhance synaptic plasticity (Trakhtenberg and Goldberg, 2012; Cho and Cavalli, 2014; Demyanenko et al., 2021). Additionally, pathway enrichment analysis has further clarified the biological processes impacted by electroacupuncture, revealing significant associations with metabolic pathways, including Lipolysis in Adipocytes and the Sphingolipid Signaling Pathway. These findings suggest that electroacupuncture may enhance metabolic resilience in the ischemic brain, potentially mitigating the energy deficits typically observed following stroke. The relationship between energy metabolism and neuronal survival is well established, as energy failure is a primary contributor to neuronal death under ischemic conditions (Sims and Muyderman, 2010; Yang et al., 2018). Furthermore, studies indicate that histone deacetylase (HDAC) enzymes play a significant role in modulating metabolic pathways, a finding that holds particular relevance in the context of ischemic stroke (Qureshi and Mehler, 2010). Therefore, understanding how electroacupuncture modulates these pathways can lead to the identification of effective therapeutic strategies that reduce oxidative stress, restore metabolic balance and support neuronal recovery. The results of this analysis not only highlight the importance of axon guidance and metabolic pathways in stroke recovery but also suggest that interventions targeting these pathways may enhance the efficacy of current treatment modalities, ultimately improving poststroke outcomes.

The association between core transcriptional circuits and super-enhancers in ischemic stroke is a critical area of investigation. Recent studies have highlighted that super-enhancers, which are large clusters of transcriptional enhancers, play a pivotal role in regulating genes involved in cellular identity and disease states (Hnisz et al., 2013; Whyte et al., 2013). Core transcription factors dominate the process of controlling gene expression programs, influencing cell states and identities. They bind not only to their own genes but also to the genes of other transcription factors, forming interconnected self-regulatory circuits known as CRCs. The core transcription factors that form CRCs are regulated by highly acetylated super-enhancers, which bind not only to their own super-enhancers but also to those associated with other important genes, thereby affecting the gene expression programs and transcription patterns of the entire cell and promoting transcriptional abnormalities in diseases. Specifically, core transcription factors facilitate gene expression by forming complexes with super-enhancers to jointly regulate gene expression (Boyer et al., 2005; Saint-Andre et al., 2016; Lambert et al., 2018; Feng et al., 2023). In the context of ischemic stroke, the dysregulation of these super-enhancers could lead to aberrant gene expression, contributing to the pathophysiology of the condition. Our findings suggest that core transcription factors such as SOX8, KLF13, and FOXK1 may serve as crucial therapeutic targets for electroacupuncture in ischemic stroke. Previous research has indicated that these transcription factors are abundantly expressed after ischemic stroke and interact with HDACs to regulate oxidative stress responses and pathological changes poststroke (Sweet et al., 2018; Jiang et al., 2022). SOX8 factors are integral to the regulation of genes associated with neuroprotection and recovery following ischemic events (Henninger and Mayasi, 2019). We hypothesized that electroacupuncture may exert its therapeutic effects by influencing these core transcription factors, thereby regulating the expression of genes driven by super-enhancers that are implicated in ischemic stroke. However, further experimental validation is needed to substantiate these findings and elucidate the precise mechanisms by which electroacupuncture interacts with core transcriptional circuits in the context of ischemic stroke.

**Figure 7:**
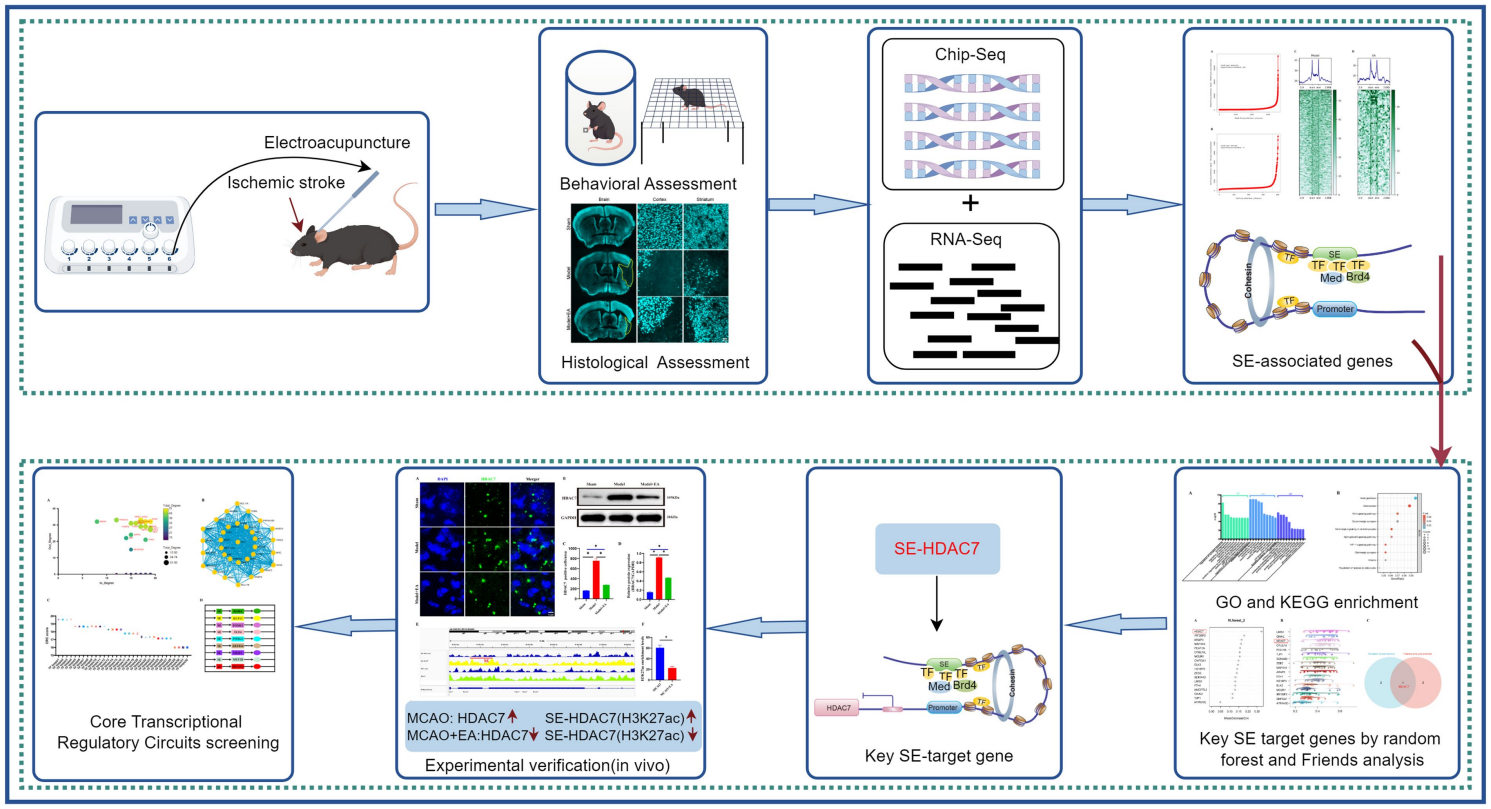
A comprehensive schematic diagram. The figure provides a comprehensive schematic diagram illustrating the research methods and findings of the entire study. Initially, the study validates the efficacy of electroacupuncture intervention in ischemic stroke by examining animal behavior and pathological changes. Brain tissues from MCAO mice subjected to electroacupuncture were analyzed using ChIP-seq and RNA-seq to investigate potential molecular regulatory mechanisms. By integrating ChIP-seq and RNA-seq data, SE-related genes were preliminarily identified, and core super-enhancer-targeted genes (SE-HDAC7) were selected as potential targets for electroacupuncture in modulating ischemic stroke, utilizing random forest machine learning and Friend analysis. Subsequent preliminary validation through immunofluorescence and immunoblotting demonstrated that electroacupuncture exerts an inhibitory effect on HDAC7 and SE-HDAC7 in ischemic stroke. Finally, potential core transcriptional regulatory circuits were explored using machine learning techniques, laying the groundwork for further in-depth research on core transcriptional circuits.

In conclusion, the potential regulatory mechanisms by which electroacupuncture may influence gene expression in the context of ischemic stroke, particularly through the modulation of super-enhancers and their associated transcriptional pathways, were elucidated in this study. The identification of key transcription factors and specific super-enhancer target genes not only enhances our understanding of the underlying biological processes but also lays the groundwork for future therapeutic strategies. As such, these findings hold promise for advancing the field of stroke treatment, although further validation and exploration are needed to fully elucidate the clinical potential of electroacupuncture (Figure 7).

## Data Availability

The datasets generated and analyzed in this manuscript are available from the Genome Sequence Archive (GSA) repository (https://ngdc.cncb.ac.cn/gsa) with the primary accession code CRA016057 and CRA020133.

## Footnotes

^1^ https://bitbucket.org/young_computation/rose

^2^ https://imagej.net/software/fiji/

^3^ https://cran.r-project.org/
