# Supplementary material for: Bioinformatics analysis of electroacupuncture treatment for ischemic stroke: exploring transcriptional regulatory mechanisms mediated by super-enhancers
Source: Front Neurosci. 2025 Mar 5;19:1522466. doi: 10.3389/fnins.2025.1522466 (PMC11920576; doi:10.3389/fnins.2025.1522466)
Supplement: Supplementary file 1 [file Data_Sheet_1.docx]

**Supplementary file Online Content**

[**Supplementary Table S1 and Figure S1: Comprehensive criteria for determining the success of the MCAO model, along with the Longa Score for each group 2**](#_Toc186708561)

[**Supplementary Table S2: The information of 18 super-enhancer-driven differential genes 3**](#_Toc186708562)

[**Supplementary Table S3: All CRCs of ischemic stroke 4**](#_Toc186708563)

# Supplementary Table S1 and Figure S1: Comprehensive criteria for determining the success of the MCAO model, along with the Longa Score for each group

Table S1: Criteria for determining the success of the MCAO model (Longa Score Scale)

| Score | Describe |
| --- | --- |
| 0 | no neurological deficit |
| 1 | retracts left forepaw when lifted by the tail |
| 2 | circles to the left while walking; |
| 3 | falls to the left or limped |
| 4 | unable to walk spontaneously; |
| 5 | dead |

Note: a score ranging from 1 to 3 points are selected for inclusion in the study for further research

Figure S1. The Longa Score for each group


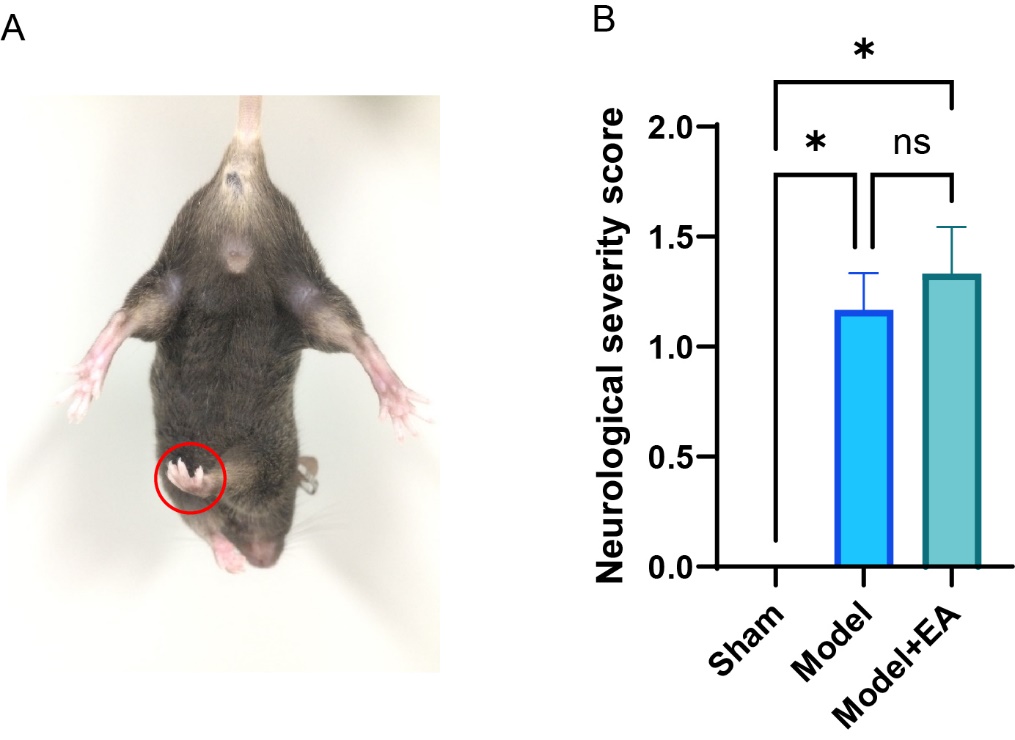


Figure S1. The Longa Score for each group

Note: Figure S1-(A). Schematic diagram of successful modeling of MCAO in mice. (B) Statistical chart of neurological function deficit scores for each group after modeling.

# Supplementary Table S2: The information of 18 super-enhancer-driven differential genes

Table1 The information of 18 super-enhancer-driven differential genes

| **Chr** | **Start** | **End** | **Annotation** | **Distance to TSS** | **Nearest Promoter ID** | **Entrez ID** | **Nearest Unigene** | **Nearest Refseq** | **Nearest Ensembl** | **Gene Name** | **tag** |
| --- | --- | --- | --- | --- | --- | --- | --- | --- | --- | --- | --- |
| chr13 | 43517349 | 43531540 | Intergenic | 35747 | NM_001081059 | 76137 | Mm.426290 | NM_001081059 | ENSMUSG00000021371 | Mcur1 | 8713.888 |
| chr7 | 65409255 | 65421695 | Intergenic | -44231 | NM_009386 | 21872 | Mm.4342 | NM_009386 | ENSMUSG00000030516 | Tjp1 | 8415.0924 |
| chr15 | 97811495 | 97826562 | Intron(NM_001204275, intron 1 of 24) | -4165 | NM_001204281 | 56233 | Mm.384027 | NM_019572 | ENSMUSG00000022475 | Hdac7 | 8790.6712 |
| chr10 | 58368685 | 58383586 | Intron (NM_201242, intron 1 of 9) | 4681 | NM_201242 | 110829 | Mm.57734 | NM_026148 | ENSMUSG00000019920 | Lims1 | 8643.16 |
| chr18 | 38366038 | 38385383 | Intergenic | -36717 | NM_011937 | 26384 | Mm.22374 | NM_011937 | ENSMUSG00000052102 | Gnpda1 | 9618.8312 |
| chr1 | 172200177 | 172217248 | Intergenic | -1908 | NM_011063 | 18611 | Mm.544 | NM_011063 | ENSMUSG00000013698 | Pea15a | 10439.528 |
| chr2 | 33220758 | 33245910 | intron (NM_011923, intron 2 of 4) | 17373 | NM_011923 | 26360 | Mm.208919 | NM_011923 | ENSMUSG00000004105 | Angptl2 | 12060.8635 |
| chr18 | 38083086 | 38100613 | Intergenic | -92880 | NM_001205336 | 106952 | Mm.247642 | NM_139206 | ENSMUSG00000024451 | Arap3 | 12160.9264 |
| chr19 | 9964264 | 9993612 | Intergenic | -1662 | NR_073181 | 14319 | Mm.1776 | NM_010239 | ENSMUSG00000024661 | Fth1 | 14439.708 |
| chr8 | 126662538 | 126695636 | Intergenic | -85101 | NM_001164598 | 270110 | Mm.334918 | NM_001164598 | ENSMUSG00000051495 | Irf2bp2 | 15761.7438 |
| chr10 | 93250241 | 93281282 | intron (NM_013508, intron 2 of 4) | 45398 | NM_001322265 | 13713 | Mm.4454 | NM_013508 | ENSMUSG00000008398 | Elk3 | 16638.512 |
| chr9 | 107611746 | 107644978 | intron (NM_008138, intron 1 of 8) | 6981 | NM_008138 | 14678 | Mm.196464 | NM_008138 | ENSMUSG00000032562 | Gnai2 | 17895.9705 |
| chr12 | 8323971 | 8351510 | exon (NM_021429, exon 6 of 7) | 24307 | NM_021429 | 58240 | Mm.309954 | NM_021429 | ENSMUSG00000020605 | Hs1bp3 | 18220.464 |
| chr13 | 51750517 | 51784452 | intron (NM_001281880, intron 1 of 16) | 26263 | NM_001281880 | 20354 | Mm.33903 | NM_013660 | ENSMUSG00000021451 | Sema4d | 18841.2672 |
| chr1 | 39919335 | 39962976 | intron (NM_001252200, intron 2 of 31) | 40242 | NM_001252200 | 26921 | Mm.491139 | NM_008696 | ENSMUSG00000026074 | Map4k4 | 23859.0814 |
| chr15 | 27753532 | 27808837 | intron (NM_001081302, intron 34 of 56) | -99642 | NM_198301 | 223433 | Mm.266485 | NM_198301 | ENSMUSG00000056069 | Otulinl | 31474.6446 |
| chr17 | 26595359 | 26663873 | exon (NM_026170, exon 5 of 10) | -46780 | NM_025272 | 11974 | Mm.22602 | NM_025272 | ENSMUSG00000015575 | Atp6v0e | 37231.051 |
| chr2 | 45032062 | 45117849 | intron (NM_001355289, intron 3 of 10) | 35322 | NM_001289521 | 24136 | Mm.440702 | NM_015753 | ENSMUSG00000026872 | Zeb2 | 58395.8916 |

# Supplementary Table S3: All CRCs of ischemic stroke

Table 2 All CRCs of ischemic stroke

| **CRC rank** | **TF list of CRCs** | **CRC score** | **TF number of CRC** |
| --- | --- | --- | --- |
| 1 | TF Loops 1: [RARA, KLF13, TCF4, FOXG1, ZBTB16, NR2F1, FOXK1, MEF2D, HIVEP2] | 19.55555556 | 9 |
| 2 | TF Loops 2: [RARA, KLF13, SMAD3, FOXG1, ZBTB16, NR2F1, FOXK1, MEF2D, HIVEP2] | 19.55555556 | 9 |
| 3 | TF Loops 3: [SOX8, KLF13, TCF4, FOXG1, ZBTB16, NR2F1, FOXK1, MEF2D, HIVEP2] | 19.55555556 | 9 |
| 4 | TF Loops 4: [SOX8, KLF13, SMAD3, FOXG1, ZBTB16, NR2F1, FOXK1, MEF2D, HIVEP2] | 19.55555556 | 9 |
| 5 | TF Loops 5: [RARA, KLF13, TCF4, FOXG1, ZBTB16, NR2F1, FOXK1, MEF2D, KLF7] | 18.66666667 | 9 |
| 6 | TF Loops 6: [RARA, KLF13, SMAD3, FOXG1, ZBTB16, NR2F1, FOXK1, MEF2D, KLF7] | 18.66666667 | 9 |
| 7 | TF Loops 7: [RARA, NFIX, NR2F1, MEF2D, TCF4, ZBTB16, FOXG1, FOXK1, HIVEP2] | 18.66666667 | 9 |
| 8 | TF Loops 8: [RARA, NFIX, NR2F1, MEF2D, SMAD3, ZBTB16, FOXG1, FOXK1, HIVEP2] | 18.66666667 | 9 |
| 9 | TF Loops 9: [SOX8, KLF13, TCF4, FOXG1, ZBTB16, NR2F1, FOXK1, MEF2D, KLF7] | 18.66666667 | 9 |
| 10 | TF Loops 10: [SOX8, KLF13, SMAD3, FOXG1, ZBTB16, NR2F1, FOXK1, MEF2D, KLF7] | 18.66666667 | 9 |
| 11 | TF Loops 11: [SOX8, NFIX, NR2F1, MEF2D, TCF4, ZBTB16, FOXG1, FOXK1, HIVEP2] | 18.66666667 | 9 |
| 12 | TF Loops 12: [SOX8, NFIX, NR2F1, MEF2D, SMAD3, ZBTB16, FOXG1, FOXK1, HIVEP2] | 18.66666667 | 9 |
| 13 | TF Loops 13: [RARA, KLF13, TCF4, PKNOX2, NR2F1, HIVEP2, MEF2D] | 18.28571429 | 7 |
| 14 | TF Loops 14: [RARA, KLF13, SMAD3, PKNOX2, NR2F1, HIVEP2, MEF2D] | 18.28571429 | 7 |
| 15 | TF Loops 15: [SOX8, KLF13, TCF4, PKNOX2, NR2F1, HIVEP2, MEF2D] | 18.28571429 | 7 |
| 16 | TF Loops 16: [SOX8, KLF13, SMAD3, PKNOX2, NR2F1, HIVEP2, MEF2D] | 18.28571429 | 7 |
| 17 | TF Loops 17: [RARA, NFIX, NR2F1, MEF2D, TCF4, ZBTB16, FOXG1, FOXK1, KLF7] | 17.77777778 | 9 |
| 18 | TF Loops 18: [RARA, NFIX, NR2F1, MEF2D, SMAD3, ZBTB16, FOXG1, FOXK1, KLF7] | 17.77777778 | 9 |
| 19 | TF Loops 19: [SOX8, NFIX, NR2F1, MEF2D, TCF4, ZBTB16, FOXG1, FOXK1, KLF7] | 17.77777778 | 9 |
| 20 | TF Loops 20: [SOX8, NFIX, NR2F1, MEF2D, SMAD3, ZBTB16, FOXG1, FOXK1, KLF7] | 17.77777778 | 9 |
| 21 | TF Loops 21: [RARA, KLF13, TCF4, FOXG1, ERF, HIVEP2] | 17.33333333 | 6 |
| 22 | TF Loops 22: [RARA, KLF13, SMAD3, FOXG1, ERF, HIVEP2] | 17.33333333 | 6 |
| 23 | TF Loops 23: [SOX8, KLF13, TCF4, FOXG1, ERF, HIVEP2] | 17.33333333 | 6 |
| 24 | TF Loops 24: [SOX8, KLF13, SMAD3, FOXG1, ERF, HIVEP2] | 17.33333333 | 6 |
| 25 | TF Loops 25: [RARA, NFIX, NR2F1, MEF2D, TCF4, PKNOX2, HIVEP2] | 17.14285714 | 7 |
| 26 | TF Loops 26: [RARA, NFIX, NR2F1, MEF2D, SMAD3, PKNOX2, HIVEP2] | 17.14285714 | 7 |
| 27 | TF Loops 27: [SOX8, NFIX, NR2F1, MEF2D, TCF4, PKNOX2, HIVEP2] | 17.14285714 | 7 |
| 28 | TF Loops 28: [SOX8, NFIX, NR2F1, MEF2D, SMAD3, PKNOX2, HIVEP2] | 17.14285714 | 7 |
| 29 | TF Loops 29: [RARA, KLF13, TCF4, FOXG1, ERF, KLF7] | 16 | 6 |
| 30 | TF Loops 30: [RARA, KLF13, SMAD3, FOXG1, ERF, KLF7] | 16 | 6 |
| 31 | TF Loops 31: [SOX8, KLF13, TCF4, FOXG1, ERF, KLF7] | 16 | 6 |
| 32 | TF Loops 32: [SOX8, KLF13, SMAD3, FOXG1, ERF, KLF7] | 16 | 6 |
